# Impacts of Seasonality on Activity Budgets and Spatial Movement of Geladas (*Theropithecus gelada*) in Susgen Natural Forest, South Wollo, Ethiopia

**DOI:** 10.1155/sci5/8232143

**Published:** 2025-08-31

**Authors:** Mulugeta Gebrie Mengistu, Hussein Ibrahim Seid, Krishnagouda Shankargouda Goudar

**Affiliations:** Department of Biology, School of Bioscience and Technology, College of Natural Sciences, Wollo University, Dessie, Ethiopia

**Keywords:** activity budget, gelada, habitat, home range, Susgen Natural Forest

## Abstract

The gelada (*Theropithecus gelada*), Ethiopia's only endemic primate and the last surviving graminivorous cercopithecid, was studied in Susgen Natural Forest, South Wollo, to examine seasonal variations in activity budgets and ranging ecology. From February to August 2023, encompassing both dry and wet seasons, 3519 behavioral scans were collected from 1680 group observations using instantaneous scan sampling at 15-min intervals (07:00–17:00 h). Data were analyzed with descriptive statistics and nonparametric tests (Kruskal–Wallis *H* and Mann–Whitney *U*), while home ranges were mapped via minimum convex polygon (MCP) and kernel density estimation (KDE). Results revealed that geladas allocated 43.2% of their time to feeding, 15% to movement, 15.5% to social activities, 13.1% to resting, and 13.2% to other behaviors. Dry seasons elicited significantly greater feeding effort (46.1% vs. 40.4%; *p* < 0.05) and daily travel distances (3658.4 ± 0.902 m vs. 3132.1 ± 2.367 m in wet season; Mann–Whitney *U*, *p* ≤ 0.05), with home ranges analyzed through the MCP method expanding to 190.1 ha in dry season as compared with 118.18 ha in wet season. KDE analysis identified the intensive use of core areas (54 ha) within broader ranges (164.95 ha). These findings underscore how geladas in human-modified landscapes face chronic nutritional stress, adapting through extended foraging and ranging patterns. We recommend immediate conservation measures, including habitat restoration and buffer zone establishment, to mitigate anthropogenic pressures on this threatened endemic species.

## 1. Introduction

Ethiopia represents a globally significant biodiversity hotspot, harboring an exceptional array of species due to its unique biogeographical characteristics and extreme topographical variation [1]. The country's remarkable ecological diversity stems from its dramatic altitudinal gradients, ranging from 125 m below the sea level in the Danakil depression to 4620 m at Ras Dejen, coupled with complex climatic patterns influenced by the Great Rift Valley and the Ethiopian Highlands [2]. This heterogeneous landscape has fostered high levels of endemism across multiple taxa, with approximately 20% of Ethiopia's mammal species and 12% of its avifauna being endemic [3, 4]. The high-altitude Afroalpine and sub-Afroalpine ecosystems (above 3200 m), characterized by moorlands and grasslands, serve as critical refugia for numerous endemic species [5, 6]. These include iconic taxa such as the Ethiopian wolf (*Canis simensis*) and the mountain nyala (*Tragelaphus buxtoni*). Concurrently, the moist evergreen montane forests of the south-western highlands support distinct endemic communities, including several primate species and the recently described Prince Ruspoli's turaco (*Menelikornis ruspolii*) [7]. Notably, even the arid lowland regions of the Somali-Masai biome in southern Ethiopia harbor unique assemblages of xeric-adapted endemics [8]. This extraordinary concentration of endemic biodiversity, occurring across multiple elevational zones and ecological systems, underscores Ethiopia's importance as a priority conservation area within the Eastern Afromontane biodiversity hotspot [9]. Primates represent one of the most extensively studied mammalian orders due to their high visibility in forest ecosystems, distinctive morphological features facilitating species identification, and their significance as model organisms for understanding behavioral ecology and evolutionary biology [10, 11]. The gelada (*Theropithecus gelada*), Ethiopia's only endemic primate and the sole extant representative of its genus, occupies a unique ecological niche in the country's highland ecosystems [12–14]. This specialized grazer predominantly inhabits the Afroalpine grasslands of Ethiopia's northwestern and central highlands at elevations between 1800 and 4400 m, with its current distribution restricted to fragmented populations across northern Ethiopia [13]. While the largest populations persist in Simien Mountains National Park, a UNESCO World Heritage Site [15], smaller, increasingly isolated groups occur in Guassa [12], Wollo (this study area), and Debre Libanos [16].

As the last surviving graminivorous primate, geladas exhibit unique behavioral and ecological adaptations to their high-altitude grassland habitats [12, 17]. Their activity budgets and ranging patterns demonstrate pronounced seasonal variations tied to resource availability, with individuals spending 60%–70% of daylight hours foraging on graminoids [12]. These patterns are further mediated by complex social structures and demographic factors across age–sex classes [18]. However, growing anthropogenic pressures—including agricultural expansion, livestock grazing, and climate change—are increasingly disrupting these natural behavioral patterns [19, 20], potentially threatening the species' long-term viability.

This study investigates the behavioral ecology and ranging patterns of geladas (*Theropithecus gelada*) in Susgen Natural Forest (SNF), with primary objectives to analyze seasonal and demographic variations in activity budgets across different age–sex classes, focusing on feeding, movement, resting, and social behaviors and to quantify ranging patterns and spatial analyses using both minimum convex polygon (MCP) and kernel density estimation (KDE) methods. The findings will contribute to evidence-based management strategies for this threatened, endemic primate species in Ethiopia's rapidly changing highland ecosystems.

## 2. Materials and Methods

### 2.1. Study Area

**Figure 1:**
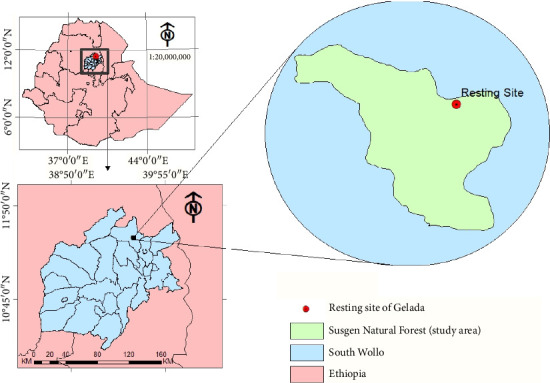
Map of the study area, Susgen Natural Forest.

SNF is located in Ambassel district, South Wollo Zone, Amahara Regional State, nearby West of Ysma-Ngus, which is a historical place of Wuchale treaty between Italia and Ethiopia. SNF is located at 11°26′53.7″ to 11°29′2.43″ latitude North and 39°32′6 12.6″ to 39°34′27.7″ longitude East with elevation ranging from 1200 to 3200 masl (Figure 1).

The mean annual rainfall of the study area was 1089.69 mm and has a bimodal type of rainfall distribution. The study area exhibited a mean annual maximum temperature of 23.52°C and a minimum of 9.95°C, resulting in an average annual temperature of 16.7°C.

According to Workayehu et al.'s [21] floristic composition, the predominant natural vegetation in the study area is classified as *Juniperus procera* forest or “dry single dominant Afromontane Forest” primarily comprising *Juniperus procera* and/or *Olea Africana* as dominant tree species.

Field observations and direct communications confirmed the presence of several mammalian species within the study area, including golden jackal (*Canis aureus*), vervet monkey (*Chlorocebus aethiops*), rock hyrax (*Procavia capensis*), porcupine (*Hystrix cristata*), spotted hyena (*Crocuta crocuta*), common duiker (*Sylvicapra grimmia*), colobus monkey (*Colobus guereza*), and gelada (*Theropithecus gelada*). In addition, diverse small mammals and avian species were coexisting in this study area alongside prevailing biotic and abiotic factors.

### 2.2. Preliminary Survey, Sampling Design, and Data Collection

This study was conducted from February 2023 to August 2023. Preliminary surveys were conducted during the first week of February to identify study sites, and habitat types were classified. The study site was systematically selected to represent distinct habitat types, stratified by altitudinal gradients and dominant vegetation composition. During this period, the distribution of geladas, climate, vegetation cover, fauna, topography, infrastructure, accessibility, organization, logistic support, hiring assistants, and gelada occupancy of the study area were assessed. A band of multimale and multifemale with a group composition of male, female, and young geladas were selected for scan sampling to quantify daily activity budgets and characterize ranging patterns within their habitat. The study band was partially habituated to human observer during the second and third weeks of February by following the group throughout the day and able to approach gently to the geladas by wearing the same cloths without recording any data. Field data collection was conducted during representative periods of both dry (late February through April) and wet (June through August) seasons to capture seasonal variation.

The actual data collection was conducted during the last week of February, March, and April for dry seasons and June, July, and August for wet seasons. Behavioral data of geladas were collected by direct observation and silent detection throughout the day. Quantitative and qualitative data on the diurnal behavioral activity time budget (ATB) and ranging ecology of geladas were gathered using the instantaneous scan sampling method outlined by Altmann [22], during both the wet and dry seasons. Behavioral data of the geladas were collected using scan sampling methods at 15-min intervals on members of the study band and ATB and ranging ecology data were collected from the habituated study band of geladas [22] during the last seven days of the study months [23–25].

Behavioral data were collected using the instantaneous scan sampling method described by Altmann [22]. Activity data were collected from the members of the habituated gelada band for 5-minute duration every 15-min intervals between 7:00 and 17:00 h for the last seven consecutive study days per month [20, 23–26], and a focal band was observed using either unaided visual observations or binoculars depending on the distance between the observer and geladas [23, 24, 26–28] to maintain optimal observation distance while minimizing disturbance. Behavioral data were collected through direct focal observation of gelada individuals at the closest feasible distance to ensure accurate recording of activities. All discernible daily behaviors were systematically documented using standardized ethograms on dedicated data collection sheets.

Behavioral data were collected using the standardized scan sampling method, recording the first 5-s sustained activity observed for each gelada individual. To minimize bias, observers systematically scanned groups in randomized directions (left–right, right–left, top–bottom, or bottom–top) [29]. Each individual was classified by the age–sex category (adult male, adult female, subadult male, subadult female, or juvenile) and assigned one mutually exclusive behavioral state: feeding, moving, resting, sexual activity (including play, aggression, allogrooming, and mating), or other activities [27, 29].

During scan sampling, the geographic centroid of the gelada band was recorded at consistent 15-min intervals. To characterize the daily movement patterns and habitat use, waypoints along the group's periphery were collected while maintaining a minimum observation distance of 20 m to avoid disturbance [25, 29]. When the band initiated movement, subsequent travel routes were mapped by recording GPS coordinates at key locations. Data were collected across both wet and dry seasons.

Spatial analysis was conducted using ArcMap 10.4 to calculate daily movement distances and home range characteristics. Initial home range estimation employed the MCP approach [26, 30–32], followed by KDE to assess space utilization patterns. The KDE method generates a probability density function [33] representing the animal's utilization distribution [34–36], thereby providing detailed insights into habitat use intensity within the home range.

### 2.3. Data Analysis

Data were checked for completeness before analysis. Nonparametric tests were used to assess variations in activity budgets: the Mann–Whitney *U* test compared seasonal differences (dry vs. wet seasons), while the Kruskal–Wallis *H* test examined variations across sex/age classes. All statistical analyses were conducted in SPSS (Version 20), with two-tailed tests and a 95% confidence interval. Significance was set at *p* ≤ 0.05. Results were summarized using descriptive statistics, with percentage values and statistical interpretations presented in figures and tables.

Furthermore, to estimate the daily ranging length and home ranges areas of the gelada, GPS points recorded in UTM coordinate during both dry and wet seasons were analyzed. All shape files were projected into the UTM Adindan 37N coordinate system to ensure consistency in geospatial calculations. Home range boundaries were delineated using the MCP method [37], employing the “Convex Hull” geometry type to enclose the outermost GPS locations [25]. To further assess space-use intensity, KDE was applied using default bandwidth parameters and the Geodesic distance method. The 95% volume contours were measured as the general home range and 50% volume contours as the core home range [30, 31], reflecting areas of highest utilization. These spatial metrics were computed to facilitate a data-driven approach to conservation planning, enabling targeted strategies for gelada populations across the study sites. The integration of MCP and KDE methods ensures a robust assessment of ranging behavior, accounting for both spatial extent and habitat-use intensity.

## 3. Results

### 3.1. Seasonal Variation in the ATB

Over a 42-day study period, a total of 3519 individual behavioral observations were recorded from 1680 group scans. The overall activity budget of geladas was dominated by feeding (43.2%), followed by resting (13.1%), moving (15.0%), and social activities (15.5%) and in other behaviors (15.2%). Geladas spent more time for feeding during the dry season (46.1%) compared with the wet season (40.4%) though this difference was not statistically significant (Mann–Whitney *U* test, *p* > 0.05). In contrast, resting was significantly (*p* ± 0.05) more frequent in the wet season (15.7%) than in the dry season (10.5%). Movement activity also varied seasonally, with geladas engaging in more locomotion during the dry season (18.3%) than the wet season (11.6%), which was statistically significant (*p* ≤ 0.05). Social activities showed a trend toward higher engagement in the wet season (17.7%) compared with the dry season (13.2%), but this significant was not significant (*p* ± 0.05). Similarly, other activities occupied more time in the wet season (14.6%) than in the dry season (11.9%), though this variation also lacked statistical significance (*p* > 0.05) as indicated in (Table 1).

### 3.2. Monthly and Demographic Variation in Gelada Behavioral Activities

**Figure 2:**
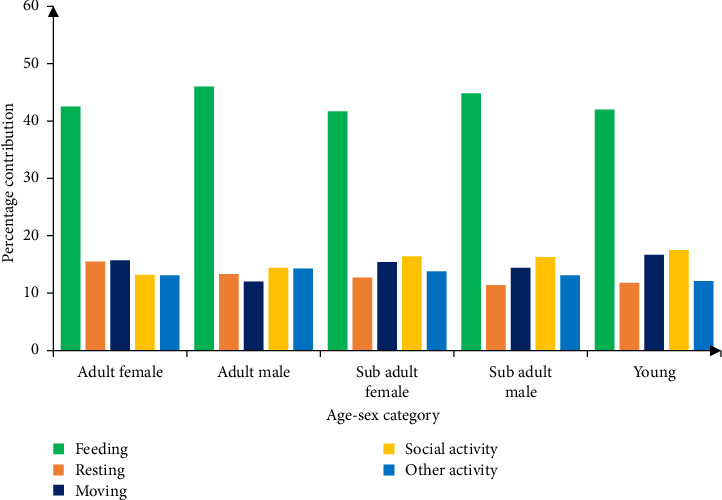
The overall behavioral activities of geladas in different age–sex category.

Feeding activity in geladas exhibited monthly variation, ranging from 50.7% in February (the highest) to 37.4% in August (the lowest). Similarly, the movement activity peaked in April (22.0%) and was lowest in June (9.7%), while resting behavior was most frequent in July (16.6%) and least frequent in April (9.3%). Social activities showed highest engagement in August (19.8%) and the lowest during April (10.2%). However, the Kruskal–Wallis *H* test revealed no significant difference in the time allocation across these behaviors over the 6-month study period (*p* > 0.05) as indicated in (Figure 2).

The overall average age–sex category of geladas engaged in different behavioral activities were 26.65% adult female, 15.9% adult male, 19.85% subadult female, and 16.95% subadult male and 20.65% were young. These proportions remained consistent across observed daily activities throughout the study as indicated in (Figure 2).

### 3.3. Seasonal Variation in Age–Sex Class Participation in Behavioral Activities

Analysis of seasonal activity patterns across age–sex classes revealed the following distributions. Adult females constituted 27.5% of the observations during the dry season and 25.8% during the wet season. Adult males maintained consistent representation at 15.9% in both seasons. Subadult females accounted for 20.7% (dry season) and 19.0% (wet season) of the observations, while subadult males comprised 16.8% (dry season) and 17.1% (wet season). Juveniles showed greater seasonal variation, representing 19.1% of the observations in the dry season and 22.2% in the wet season.

The Kruskal–Wallis *H* test indicated no significant seasonal differences in time allocation to behavioral activities across all age–sex classes (*p* > 0.05), suggesting consistent activity patterns regardless of seasonal variation.

### 3.4. Age–Sex Class Differences in Activity Budgets

Analysis of activity budgets across age–sex classes revealed distinct behavioral patterns (Figure 2). Feeding dominated all categories: adult females (42.5%), adult males (46.0%), subadult females (41.7%), subadult males (44.8%), and juveniles (42.0%). Resting accounted for 15.5% of adult female, 13.3% of adult male, 12.7% of subadult female, 11.4% of subadult male, and 11.8% of juvenile activity. Movement constituted 15.7% (adult females), 12.0% (adult males), 15.4% (sub adult females), 14.4% (subadult males), and 16.7% (juveniles) of time budgets. Social activities represented 13.2% of adult female, 14.4% of adult male, 16.4% of subadult female, 16.3% of subadult male, and 17.5% of juvenile time allocation. Other activities accounted for 13.1% (adult females), 14.3% (adult males), 13.8% (subadult females), 13.1% (subadult males), and 12.1% (juveniles) of the observed behaviors (Table 2).

Notably, all age–sex classes prioritized feeding during both dry and wet seasons in SNF. Most groups exhibited higher feeding and movement activity during the dry season compared with the wet season.

### 3.5. Ranging Ecology

#### 3.5.1. Seasonal Variation in the Daily Range Length of Geladas

**Figure 3:**
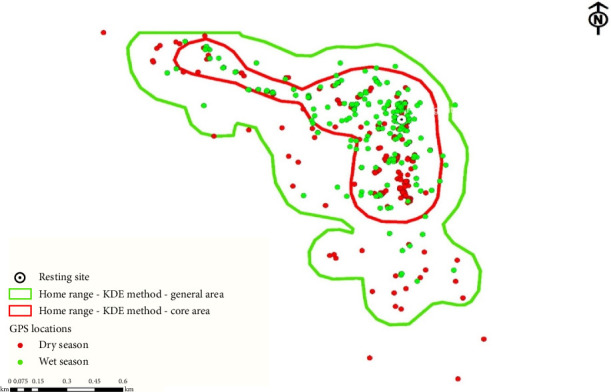
The overall general and core home range size of geladas using the KDE method.

Daily range lengths were calculated by combining GPS tracking data from complete day follows (*n* = 7 days) and analyzing coordinate points using GIS software. As shown in (Figure 3), monthly variations revealed a maximum mean daily range length in March (3790.5 m) and a minimum in August (2901.5 m).

Seasonal analysis demonstrated significant differences in movement patterns. During the 6-month study period, geladas exhibited longer daily ranges in the dry season (mean = 3658.4 m, equivalent to 3.6 km) compared with the wet season (mean = 3132.1 m, or 3.1 km), with an overall mean of 3395.25 m (Mann–Whitney *U* test, *p* ≤ 0.05). This seasonal variation was reflected in the home range size, which expanded to 190.1 ha during the dry season compared with 118.18 ha in the wet season.

Band-level movement patterns showed consistent seasonal differences, with dry season daily travel distances averaging 3658.4 ± 0.902 m (mean ± SE) versus 3132.1 ± 2.367 m during the wet season. These findings demonstrate significant seasonal plasticity in gelada ranging behavior as indicated in (Table 3).

#### 3.5.2. Home Range Estimation Using the MCP Method

**Figure 4:**
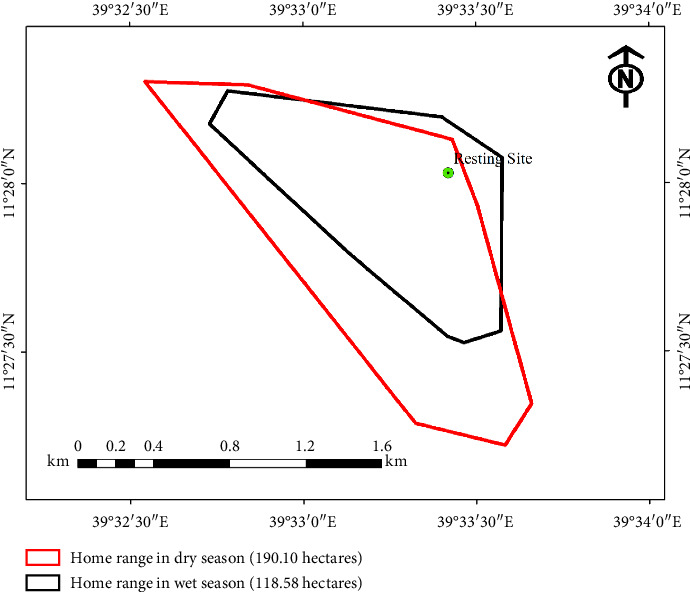
The home range size of geladas during the dry and wet seasons in SNF.

The overall home range areas of the study band, estimated using the MCP method, were 190.1 ha during the dry season and 118.18 ha during the wet season (Figure 4). This indicates a significant expansion of home range during the dry season (Mann–Whitney *U* test, *p* ± 0.05). The observed seasonal variation likely reflects differences in resource availability. During the dry season, geladas traveled longer distances and utilized a wider area, presumably due to reduced food availability within their core habitat. In contrast, the wet season provided abundant rainfall, promoting plant growth and localized food resources, which allowed the band to meet its nutritional needs within a smaller range.

#### 3.5.3. Home Range Estimation Using Kernel Density Analysis

KDE provides more reliable ranging patterns than MCP methods, revealing that geladas occupied a total home range of 164.95 ha (95% volume contour) with a core utilization area of 54.00 ha (50% volume contour), as visually represented in (Figure 3) by light green and red contour lines, respectively. This method effectively captured the intensity of habitat use, clearly differentiating between the frequently used core areas (54 ha) and the broader home range (164.95 ha), demonstrating KDE's superior ability to characterize ranging behavior compared with conventional MCP approaches.

## 4. Discussion

The study findings demonstrate that geladas allocated the majority of their activity budget to feeding (43.2%) and locomotion (15%), with significantly increased time investment during the dry season (feeding: 46.1% and movement: 18.3%). This behavioral pattern suggests potential resource limitation within their habitat, likely resulting from multiple anthropogenic and ecological pressures such as habitat encroachment by human settlements and agricultural expansion, resource competition with domestic livestock, seasonal fluctuations in food availability, and quality and degradation of native grasslands. The increase in feeding and movement effort, particularly during dry periods, reflects adaptive responses to these ecological constraints. These findings align with previous reports from disturbed habitats [24] but contrast with studies of populations in protected areas [32], highlighting the significant impact of human activities on gelada behavioral ecology.

This study demonstrates that geladas experience significant food insecurity, particularly regarding protein and energy sources during the dry season. This nutritional stress is reflected in their activity budget, with feeding dominating their time allocation (43.2% overall, increasing to 46.1% in dry seasons) at the expense of resting and social activities. This pattern contrasts markedly with Dunbar [33] observations of populations in optimal habitats where geladas could meet nutritional requirements with less foraging effort. The seasonal increase in feeding time (dry: 46.1% vs. wet: 40.4%) coincides with reduced availability and quality of preferred grass species, which become desiccated or are outcompeted by livestock grazing. These ecological constraints force geladas to either expand their dietary breadth or invest additional time searching for adequate nutrition, mirroring findings from other disturbed habitats [24, 32]. Such behavioral adjustments, while adaptive in the short term, may have long-term consequences for population viability in this already geographically restricted species. The results reveal significant seasonal variation in gelada activity budgets, with increased movement (dry season: 18.3% vs. wet season: 11.6%) and reduced resting time (dry: 10.5% vs. wet: 15.7%) during periods of resource scarcity. This pattern likely reflects the need to travel greater distances to locate adequate food and water in the dry season when natural vegetation is sparse and competition with livestock intensifies. Conversely, during wet seasons, improved forage availability and potential human-imposed movement restrictions near agricultural areas appear to reduce ranging requirements. These findings align with observations from multiple disturbed habitats [17, 23, 34] but contrast with data from protected areas like Guassa Community Conservation Area (GCCA) [16, 32], where lower foraging pressure allows for different activity allocations. The observed patterns underscore how anthropogenic landscape modification fundamentally alters gelada ranging ecology and energy budgets. Our findings demonstrate pronounced temporal variation in gelada behavioral patterns, shaped by a combination of ecological constraints and life history demands. The consistent prioritization of feeding across all age–sex classes throughout the study period suggests persistent nutritional challenges in this population. This pattern appears particularly acute among reproductive females, who exhibited elevated feeding time during dry seasons, likely reflecting the increased metabolic demands of lactation [17, 33]. Such behavioral adjustments highlight the population's adaptive responses to environmental stressors.

The observed activity patterns emerge from multiple interacting ecological pressures. Resource competition with domestic livestock [35] has substantially reduced the availability of preferred grass species, forcing geladas to either expand their foraging ranges or diversify their diets. Simultaneously, climatic variability, including bimodal rainfall patterns and extreme weather events [23], creates unpredictable fluctuations in food availability. These natural challenges are compounded by anthropogenic disturbances, particularly agricultural encroachment and retaliatory killings [13, 24, 27, 32, 36], which further fragment habitats and disrupt natural ranging behaviors.

Notably, the population's behavioral profile differs markedly from those in protected areas such as GCCA [32]. While our study population allocates substantial time to feeding year-round (dry season: 46.1% and wet season: 40.4%), geladas in less disturbed habitats can afford greater investment in resting and social activities. This contrast underscores how human-modified landscapes alter fundamental aspects of gelada behavioral ecology, potentially pushing populations toward their metabolic limits with significant implications for long-term viability.

The persistent nutritional stress evident in our findings suggests this population operates under chronic ecological constraints. The need to maintain high feeding effort across all seasons, even during periods of theoretical abundance, indicates potential carrying capacity issues in this fragmented habitat. These results have important conservation implications, particularly for managing human–wildlife conflicts and preserving critical resources in gelada habitats.

Analysis of ranging behavior provides critical insights into ecological relationships and habitat quality [38]. Our study revealed significant seasonal variation in gelada movement patterns, with mean daily range lengths extending to 3658.4 m in during dry seasons compared with 3132.1 m in wet periods. This expansion of home range size during resource-scarce conditions (dry season: 190.1 ha vs. wet season: 118.18 ha) reflects adaptive responses to multiple ecological pressures.

Methodological approaches yielded complementary perspectives on habitat use. While MCP analysis indicated a total range of 2.122 km^2^, KDE provided more biologically meaningful measures, identifying core (0.054 km^2^) and general (1.65 km^2^) utilization areas. These results align with established ecological principles that link ranging patterns to resource distribution [39, 40], while demonstrating KDE's superiority in detecting intensity of habitat use compared with MCP's perimeter-based approach.

The observed seasonal shifts mirror findings from Wonchit Valley [24] but exceed their reported distances, suggesting greater habitat degradation in our study area. During wet seasons, geladas concentrated movements in core areas with adequate forage, while dry season resource depletion forced expansion into peripheral zones—a pattern consistent with Snyder-Mackler et al.'s [18] observations of patchy resource utilization. This ranging plasticity occurs despite significant anthropogenic constraints, including livestock competition reducing forage quality in core habitats, agricultural encroachment eliminating buffer zones, and seasonal human activity fluctuations altering access patterns.

Notably, even core areas showed evidence of disturbance, with geladas frequently venturing into agricultural lands during dry periods. These findings emphasize how habitat fragmentation compounds natural seasonal challenges, forcing energy-intensive behavioral adaptations that may impact population viability. Our spatial analyses provide essential baseline data for conservation planning, particularly regarding boundary demarcation and habitat restoration priorities.

## 5. Conclusion

This study reveals that geladas in SNF exhibit significant behavioral and spatial adaptations to ecological pressures, allocating 43.2% of their activity budget to feeding and expanding their daily range to 3658.4 m (home range: 190.1 ha) during dry seasons. These patterns, coupled with KDE results (core area: 54 ha and general range: 164.95 ha), reflect higher nutritional stress and severe seasonal resource scarcity. The pronounced dry-season increases in feeding effort (46.1%) and ranging behavior contrast markedly with protected populations, highlighting the urgent need for conservation interventions including habitat restoration, buffer zone establishment, and community-based management to ensure the species' persistence in Ethiopia's modified highlands.

## Figures and Tables

**Table 1:** Percentage seasonal activity time budget of geladas in Susgen Natural Forest.

Season	Month	Feeding	Resting	Moving	Social activities	Other activities
Dry	February	17.4	3.6	5.6	4.6	3.1
	March	15.3	3.9	5.6	5.3	3.5
	April	13.4	3	7.1	3.3	5.3
	**Average**	**46.1**	**10.5**	**18.3**	**13.2**	**11.9**

Wet	June	15.3	5.3	3.3	5.3	4.9
	July	12.7	5.5	3.8	5.8	4.9
	August	12.4	4.9	4.5	6.6	4.8
	**Average**	**40.4**	**15.7**	**11.6**	**17.7**	**14.6**

**Table 2:** Activity time budget of geladas based on age/sex category in SNF.

Season	Age/sex category	Activity time budget of gelada in dry and wet seasons				
		Feeding (%)	Resting (%)	Moving (%)	Social activity (%)	Other activity (%)
Dry	Adult female	47.4	12.3	16.9	11.3	12.1
	Adult male	54.1	9.6	12.5	11.3	12.5
	Subadult female	45	8.7	18.5	14.4	13.4
	Subadult male	49.5	11.1	19.5	10.8	9.1
	Young	35.8	10.1	24	18	12.1
	Total	46.1	10.5	18.3	13.2	11.9

Wet	Adult female	37.5	18.6	14.6	15.1	14.2
	Adult male	37.8	16.9	11.5	17.6	16.2
	Subadult female	38.5	16.6	12.3	18.4	14.2
	Subadult male	40	11.7	9.3	21.7	17.3
	Young	47.9	13.7	9.3	17	12.1
	Total	40.4	15.7	11.6	17.7	14.6

**Table 3:** The mean monthly daily range length of the gelada in SNF.

Season	Month	Average day range
Dry	February	3601.3
	March	3790.5
	June	3583.3
	**Average**	**3658.4 **±** 0.902**^∗^

Wet	April	3439.5
	July	3055.3
	August	2901.5
	**Average**	**3132.1 **±** 2.367**

^∗^The Mann–Whitney *U* test showed significant difference in the daily range lengths of geladas between the wet season and the dry season (*p* ≤ 0.05).

## Data Availability

The datasets used and/or analyzed during the current study are available from the corresponding author upon reasonable request.

## Nomenclature

ATB: Activity time budget
GCCA: Guassa Community Conservation Area
KDE: Kernel density estimates
MCP: Minimum convex polygon
SNF: Susgen Natural Forest
